# Identification and Plasma Diagnostics Study of Extreme Ultraviolet Transitions in Highly Charged Yttrium

**DOI:** 10.3390/atoms5030030

**Published:** 2017

**Authors:** Roshani Silwal, Endre Takacs, Joan M. Dreiling, John D. Gillaspy, Yuri Ralchenko

**Affiliations:** 1 Department of Physics and Astronomy, Clemson University, Clemson, SC 29634, USA; 2 National Institute of Standards and Technology, Gaithersburg, MD 20899, USA; 3 National Science Foundation, Arlington, VA 22230, USA

**Keywords:** highly charged ions, yttrium, spectroscopy, extreme ultraviolet, Li-like, Na-like, magnetic dipole, plasma diagnostics, electron beam ion trap, non-Maxwellian plasma

## Abstract

Extreme ultraviolet spectra of the L-shell ions of highly charged yttrium $\left({\text{Y}}^{26+}-{\text{Y}}^{36+}\right)$ were observed in the electron beam ion trap of the National Institute of Standards and Technology using a flat-field grazing-incidence spectrometer in the wavelength range of 4 nm-20 nm. The electron beam energy was systematically varied from 2.3 keV–6.0 keV to selectively produce different ionization stages. Fifty-nine spectral lines corresponding to Δn=0 transitions within the n=2 and *n* = 3 shells have been identified using detailed collisional-radiative (CR) modeling of the non-Maxwellian plasma. The uncertainties of the wavelength determinations ranged between 0.0004 nm and 0.0020 nm. Li-like resonance lines, $2s-2p_{1/2}$ and $2s-2p_{3/2}$, and the Na-like D lines, $3s-3p_{1/2}$ and $3s-3p_{3/2}$, have been measured and compared with previous measurements and calculations. Forbidden magnetic dipole (M1) transitions were identified and analyzed for their potential applicability in plasma diagnostics using large-scale CR calculations including approximately 1.5 million transitions. Several line ratios were found to show strong dependence on electron density and, hence, may be implemented in the diagnostics of hot plasmas, in particular in fusion devices.

## 1. Introduction

Multi-electron ions are under intense theoretical study as state-of-the-art calculations rival highly accurate measurements sensitive to higher order terms of quantum electrodynamics (QED) corrections to atomic energy levels [1]. While elements with a high-Z atomic number have these effects amplified, ions in the medium-Z region have special importance because they allow for more accurate experiments and provide constraints to theoretical trends. In the past few years, the electron beam ion trap (EBIT) research program at the National Institute of Standards and Technology (NIST) has reported accurate measurements in the extreme ultraviolet (EUV) region that focus on systematic observations of transitions in L-shell, M-shell and N-shell ions [2–14]. The work reported here extends these results to a range of previously unobserved transitions of a fifth row element, yttrium.

Yttrium was chosen for the current investigation because of its relevance as a possible diagnostic impurity in tokamak fusion plasmas. For instance, together with strontium, zirconium, niobium and molybdenum, yttrium has been injected into the Texas Experimental Tokamak (TEXT) [15,16], the Joint European Torus (JET) tokamak [17] and the Princeton tokamaks [18–20] and has also been observed in laser-produced linear plasmas [16,21]. L-shell ions of high-Z elements, especially Be-like to Ne-like [22–26], and a few M-shell ions such as Na-like, Mg-like and Al-like [15,16,27–30] were used to diagnose these hot plasmas for decades. The elemental abundance of yttrium in stars also makes it astronomically important. Its relevance in nuclear astrophysics, weak interaction physics and nuclear structure physics has been discussed [31–34].

Among the various transitions in these elements, special interest is devoted to forbidden transitions that originate from long-lived metastable energy levels. The importance of the forbidden transitions in medium-Z and high-Z elements has been demonstrated by different researchers for astrophysical [35] and fusion [18,36] plasmas. For example, charge states near closed shells include potentially useful forbidden transitions such as those between the 2*s*^2^2*p*^5^−2*s*2*p*^6^ configurations of F-like ions and the magnetic dipole transition 2*s*^2^2*p*^5^ ^2^*P*_3/2_−^2^*P*_1/2_ in the same ion. These have been extensively investigated in earlier studies [37–40].

There have been a few EUV measurements of highly charged yttrium over the past couple of decades. Alexander et al. observed the EUV spectra of Y IX–XIII in the wavelength range of 4.5 nm–35 nm using vacuum spark [41]. Ekberg et al. performed a series of measurements for the identification of transitions in Si-like Y XXVI [42], Al-like Y XXVII [43] and Mg-like Y XXVIII [21,28] in the EUV spectra emitted from line-focus laser-produced plasmas as part of the X-ray laser research program. Reader et al. have reported observations of F-like Y XXXI [37], Mg-like Y XXVIII [44] and Na-like Y XXVIV [16,45] using laser-produced and tokamak plasmas in a series of systematic spectroscopic studies. Similar experiments for moderate charge states also reported observations of multiply-charged yttrium spectra (Y II–XI); see, e.g., [46–50]. Despite these experiments, the second row isoelectronic sequences of yttrium have largely been unexplored in the EUV region to date.

In this paper, we report the systematic study and identification of atomic spectral lines of the L-shell charge states of yttrium ranging from Li-like to Ne-like ions (Y XXX–Y XXXVII) created and trapped in the NIST EBIT [51,52]. We also present the most pronounced spectral lines of the Na-like, Mg-like and Al-like yttrium charge states, as these can provide benchmark experimental results for precise multi-electron atomic theory calculations. Na-like D1 and D2 lines originate from quasi-hydrogenic ions and have been used as a probe of QED contributions due to their high intensities and the available precise ab initio calculations [6,53–55]. We report the first data for the wavelengths of the Na-like D1 and D2 yttrium lines measured with an EBIT to provide accurate experimental results that complement the previously reported measurements of Reader et al. [45] in laser-produced and tokamak plasmas.

In addition to the spectral analysis, we also discuss the forbidden magnetic dipole (M1) transitions of highly charged yttrium ions that are potentially important for plasma diagnostics. The spectroscopy of forbidden magnetic dipole lines can help deduce important plasma parameters such as the density and temperature of plasmas. These parameters are obtained in practice from intensity ratios of various atomic spectral lines rather than direct measurements, which are difficult or even impossible in fusion, laboratory and astrophysical plasmas [56]. The availability of accurate collisional-radiative models makes this technique a reliable tool for plasma diagnostics [57–59].

M1 transition probabilities strongly depend on the spectroscopic charge $Z_{sp}$, and for highly ionized ions, these transitions become prominent. At low electron densities, the radiative decay rates are substantially larger than collisional depopulation from both metastable and allowed excited levels. At higher densities, however, the metastable levels decay both by collision and radiation, whereas allowed transitions still take place mostly by radiative decay. This makes the ratio of the allowed versus forbidden transitions dependent on the electron density.

The following sections describe the experimental method, the theoretical calculations that aided in line identifications, the list of the observed transitions and their uncertainties and a discussion of the diagnostic capabilities of some of the M1 transitions.

## 2. Experiment

The NIST EBIT and a multi-cathode metal vapor vacuum arc ion source (MEVVA) were used to produce highly charged yttrium ions, and the ion spectra were recorded with a custom-made EUV flat field grazing incidence spectrometer [60]. Both the MEVVA and the NIST EBIT are discussed in detail elsewhere [51,61], but we will now briefly review the most important details.

The MEVVA, which produces singly-charged ions by sparking a high voltage across metal cathodes, is located ≈ 2 m above the central trapping region of the EBIT. The ions are created at a potential of about 10 kV above ground and are accelerated towards the center of the EBIT through several electrodes at lower voltages. The trapping region, consisting of the drift tubes (upper, middle and lower), is floated on top of the voltage of the cylindrically-shaped shield electrode. To capture the ions in the trap, the shield electrode voltage is very briefly (on the order of 10^−3^ s) switched to a potential of about 9.6 kV, and the middle drift tube voltage is simultaneously raised by an additional 0.4 kV. Then, precisely at the arrival of the ions, the middle drift tube is pulsed down to the shield voltage in order to trap and confine the plasma in the trap. During the entire timing sequence, the lower and upper drift tubes are kept at constant potentials with respect to the shield (0.5 kV and 0.26 kV, respectively) to create axial trapping. Radial confinement is accomplished by a combination of the axial 2.7 T magnetic field and the space charge of the intense electron beam, which is directed through the drift tubes to further ionize the ions. The electron beam energy can then be set as required for the experiment by adjusting the shield electrode voltage. The beam energy in the EBIT is determined by the voltage difference between the electron gun and the middle drift tube, taking into account the space charge of the electron beam in the interaction region [62]. The latter depends on the density of the electron beam and therefore scales with the beam energy and current in addition to the ion cloud neutralization factor, which is generally difficult to quantify. In our experiment, the modeling of the observed spectral line intensities showed that the space charge correction was approximately 150 eV. Electron beam currents were varied between 66 mA and 147 mA during the measurement. To control the charge-state distribution of the yttrium ions, the energy of the electron beam was systematically varied from 2.3 keV–6.0 keV.

The flat field EUV grazing incidence spectrometer [60] is equipped with a liquid-nitrogen-cooled charged-coupled device (CCD) detector with 2048 × 512 active pixels of 13.5 μm × 13.5 μm size each. The spectrometer consists of a gold-coated spherical focusing mirror that focuses light radiated from the EBIT plasma onto a slit, followed by a gold-coated concave reflection grating with a groove spacing of approximately 1200 lines mm^−1^. The instrument has a resolving power of λ/dλ≈400. The 2D images recorded by the CCD were hardware collapsed along the vertical axis, so that the resulting image was a 1D (2048 × 1) spectrum. Ten 60 s frames of yttrium spectra were collected in a set, giving a total acquisition time of 600 s for each energy. The spectra were filtered of cosmic rays using a program that identifies outlier intensities among different frames within the same set. If the intensity of a channel in a certain frame is five or more Poisson standard deviations away from the mean of all of the frames, it is replaced by the average value of the other frames before the frames are summed together to form the overall spectrum.

## 3. Wavelength Calibration

Spectra emitted by yttrium ions were acquired in the approximate wavelength region of 4 nm to 20 nm. Wavelength calibration was performed using highly charged neon lines (Ne V–VIII), xenon lines (Xe XLI–XLII), barium lines (Ba XLIII–XLIV), oxygen lines (O V–VI) and iron lines (Fe XXIII–XXIV) [5,7,11,63,64], as described in this section. Neon and carbon dioxide gases were injected into the EBIT as neutral atoms from the gas injection setup described by Fahy et al. [2], with the injection pressure normally on the order of 10^−3^ Pa. Iron ions were loaded from the MEVVA ion source. Small amounts of barium and xenon ions are always present in the trap as heavy ion contaminants from the electron gun and the ion pumps. In order to prevent long-term accumulation of these ions, the EBIT trap was emptied and reloaded every 10 s.

The calibration lines were fitted using unweighted Gaussian profiles, and the locations of the peaks were noted in terms of the channel (pixel) numbers corresponding to the respective lines. The literature-recommended wavelengths [5,11,64] were plotted as a function of channel number weighted with the uncertainty in these wavelengths. A third order polynomial from the fit was used to convert the uncertainties in channel number to the uncertainties in wavelength. The statistical uncertainties of the calibration lines were then determined from the quadrature sum of these uncertainties with the adopted wavelength uncertainties from the literature.

**Figure 1.**
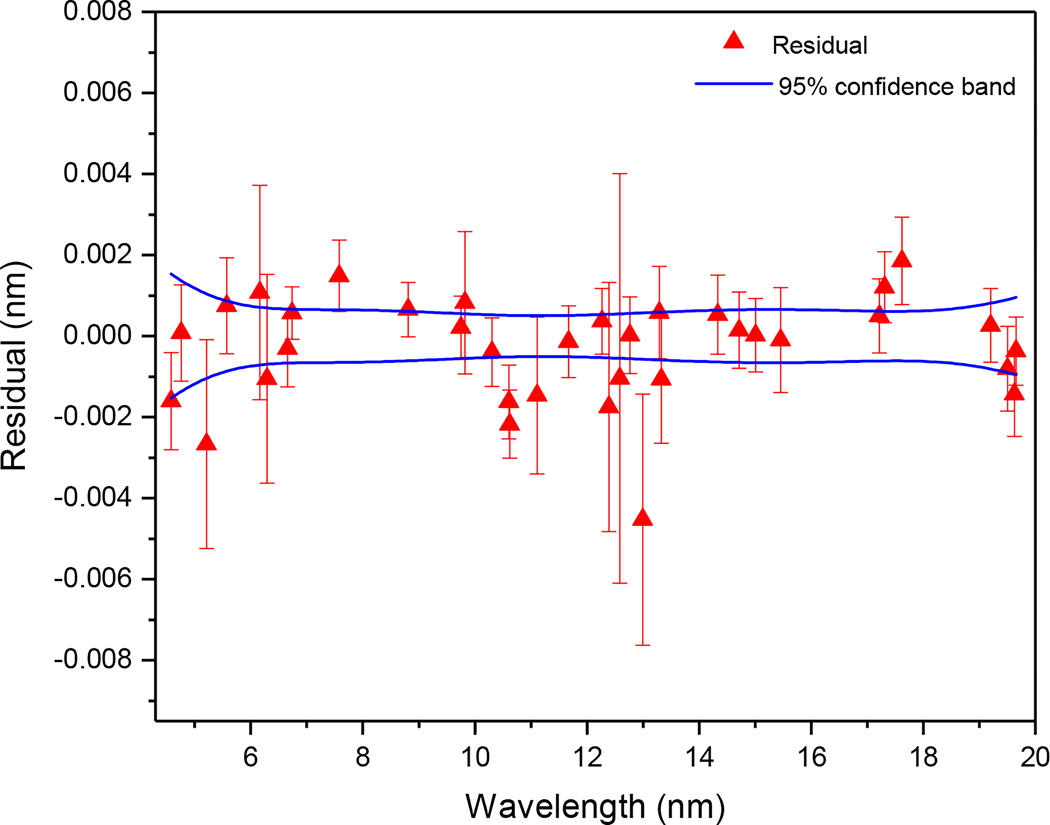
Residual of the adopted wavelength of the calibration lines with respect to their calibrated wavelength. The individual uncertainties of the data points are as described in the text. The solid (blue) line corresponds to the 95% confidence band of the calibration fit.

The final calibration function was a third order polynomial that describes the wavelength versus channel as a fit weighted by the inverse square of the total uncertainties of the lines. The latter was calculated as the quadrature sum of the overall statistical uncertainty and the systematic uncertainty. The systematic uncertainty was estimated to be 0.0006 nm by requiring the reduced chi-square of the fit to be 1 according to the standard statistical procedure [65]. Systematic uncertainty may arise from several factors during the experiment such as small device vibrations or uneven pixel response. The residual of the literature values of the calibration lines with respect to their calibrated wavelength provided an assessment of the quality of the calibration. Including their uncertainties, 95% of the residual should lie within two standard deviation (σ) of their mean (μ): P(μ−2σ≤x≤μ+2σ)≈ 0.9545. Figure 1 shows the calibration data points and 95% confidence band of the fit.

In calculating the overall calibration uncertainty contributing to the total uncertainty at a given wavelength for the identified yttrium lines, we have used the 95% confidence band at the position of the line. The calibration uncertainties reported are equal to the vertical width of the confidence band divided by four (equivalent to one standard deviation). The calibration uncertainty calculated from the 68.3% confidence band corresponding to one standard deviation gives comparable results, as expected.

## 4. Theoretical Modeling

The spectral modeling for the non-Maxwellian EBIT plasma was performed with the collisional-radiative code NOMAD [66] that has been extensively used in EBIT spectroscopy. The yttrium plasma was assumed to be in the steady state, optically thin and uniform with electron density of 10^11^ cm^−3^. The electron beam energy distribution was modeled by a Gaussian function with the full width at half maximum of 40 eV.

A detailed collisional-radiative (CR) model would generally require a large amount of atomic data, such as energy levels, wavelengths, transition probabilities and cross-sections. For the present analysis, we make use of the Flexible Atomic Code (FAC) [67], which is based on a fully-relativistic model potential and can consistently generate all required data. In total, our CR model included 13 ionization stages from Si-like to He-like ions of yttrium, about 5000 atomic levels and nearly 1.5 million transitions describing spontaneous radiative decays, electron-impact ionization and excitation, as well as radiative recombination. NOMAD also takes into account the charge exchange of ions with neutral atoms present in the trap, which shifts the ionization balance to lower charge states. Within the model, the density of neutral atoms is a free parameter and adjusted such that the theoretical and experimental spectra closely agree. The neutral densities obtained from the spectra of the current experiment are consistent with previous values under similar EBIT conditions.

Another adjustable parameter (although less important due to the lower sensitivity of the results to its variations) is the space charge correction to the electron beam energy as described in the experimental section. A generally good match between the observed and calculated line intensity ratios was obtained with a 150 eV correction to the values calculated from the applied voltages.

The CR model used these data to build and solve a system of rate equations to determine level populations and line intensities for EBIT plasmas of given electron energies. With this approach, NOMAD was used to simulate the yttrium emission as the electron beam energy was systematically changed during the experiment. The calculated spectra were convoluted with the spectrometer energy resolution and corrected for the efficiency of the grazing incidence instrument to obtain the theoretical result.

## 5. Line Identification

**Figure 2.**
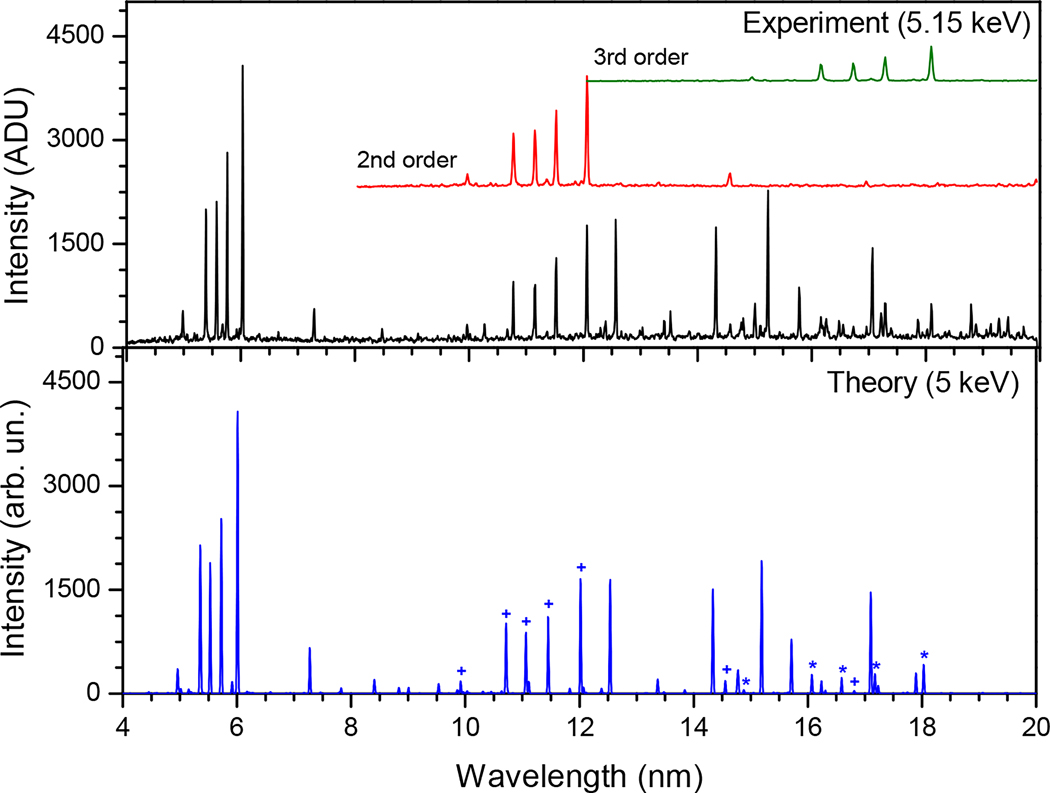
Comparison of the experimental spectrum (**top**) of yttrium with the theoretical spectrum (**bottom**). The intensity is given in analog to digital units (ADU) for the measured spectra. The second and third order spectra for the experimental data are also shown (red and green insets, respectively). The theoretical spectrum includes the second (+) and third (∗) order spectra and is calculated at an energy of 5 keV for the electron beam energy of 5.15 keV, to account for the space-charge correction in the experiment.

Yttrium spectra were taken as a function of the calibrated wavelength and fitted with unweighted Gaussians to determine the line positions. The uncertainty associated with the identified lines was then calculated from the quadrature sum of the uncertainty of the line fit that corresponds to the statistical uncertainty, the calibration uncertainty, the systematic uncertainty (estimated using the calibration data as discussed above) and uncertainty assigned for a possible small systematic line asymmetry (discussed below), which might be due to line blends or instrument asymmetries. In order to reach the desired ionization stages, the beam energy was systematically varied from 2.3 keV–6.0 keV. By matching theoretical and experimental spectra, we were able to conveniently identify most of the yttrium lines, as shown in Figure 2.

Some of the yttrium lines were also observed in the second and third orders of diffraction, in addition to the first order. Second order and third order yttrium spectra were plotted simultaneously as a visual aid to better identify the observed lines. They were obtained by dividing the line intensities of the first order experimental spectra by 2.5 and eight and multiplying the wavelength by two and three, respectively. Since we observed the same yttrium lines at several different beam energies, our reported wavelengths are the weighted averages of the positions of these lines using the formula for the best combined estimate of N measurements of the same quantity, $x_{CE}=\frac{\sum w_{i}\times x_{i}}{\sum w_{i}},x_{i}$ being the line position at different energies. The weight $w_{i}$ is given by $w_{i}=\frac{1}{s_{i}^{2}}$, where $s_{i}$ is the total uncertainty corresponding to each measurement. A few lines were blended with unresolved features, making it difficult to precisely determine their positions. In such cases, the spectra at energies that gave the cleanest and strongest signals were solely used. As a test for unanticipated systematics, the difference in the individual wavelengths at different energies with their weighted average was calculated. This difference was binned to get a histogram that represents a normal distribution about their mean, which should be zero. The distribution was fitted with a Gaussian function, and the mean value of 0.0003 nm was assigned to be the uncertainty due to unknown line asymmetries. As mentioned earlier, this uncertainty was added in quadrature to the rest of the uncertainties to get the total line uncertainty. For the lines that we observed in second and third order, the wavelengths and the corresponding uncertainties were divided by two and three, respectively, and the weighted average was calculated accordingly. The total uncertainty for each of the identified lines was computed using the error propagation method $s=\sqrt{\frac{1}{\sum \frac{1}{s_{i}^{2}}}}$.

The most prominent observed lines were unambiguously identified through comparison with theory. For instance, the Be-like lines 2*s*^2^ ^1^*S*_0_−2*s*2*p* ^1^*P*_1_ and 2*s*^2^ ^1^*S*_0_−2*s*2*p* ^3^*P*_1_ were identified at 6.0322(5) nm and 15.2336(7) nm experimentally compared to the calculated values of 6.0098 nm and 15.1907 nm. The measured wavelengths of the Li-like, B-like, C-like and N-like yttrium lines are within 1.3% of our theoretical values, sufficient for line identification purposes. We note that for electron beam energies below 3.75 keV, where M-shell yttrium ion charge states become prominent, a more accurate relativistic many-body perturbation theory (RMBPT) calculation had to be invoked to match theoretical and experimental data. These calculations were performed by Safronova et al. [68] for the lines of the Ne-like, Na-like, Mg-like and Al-like charge states of yttrium lines.

**Figure 3.**
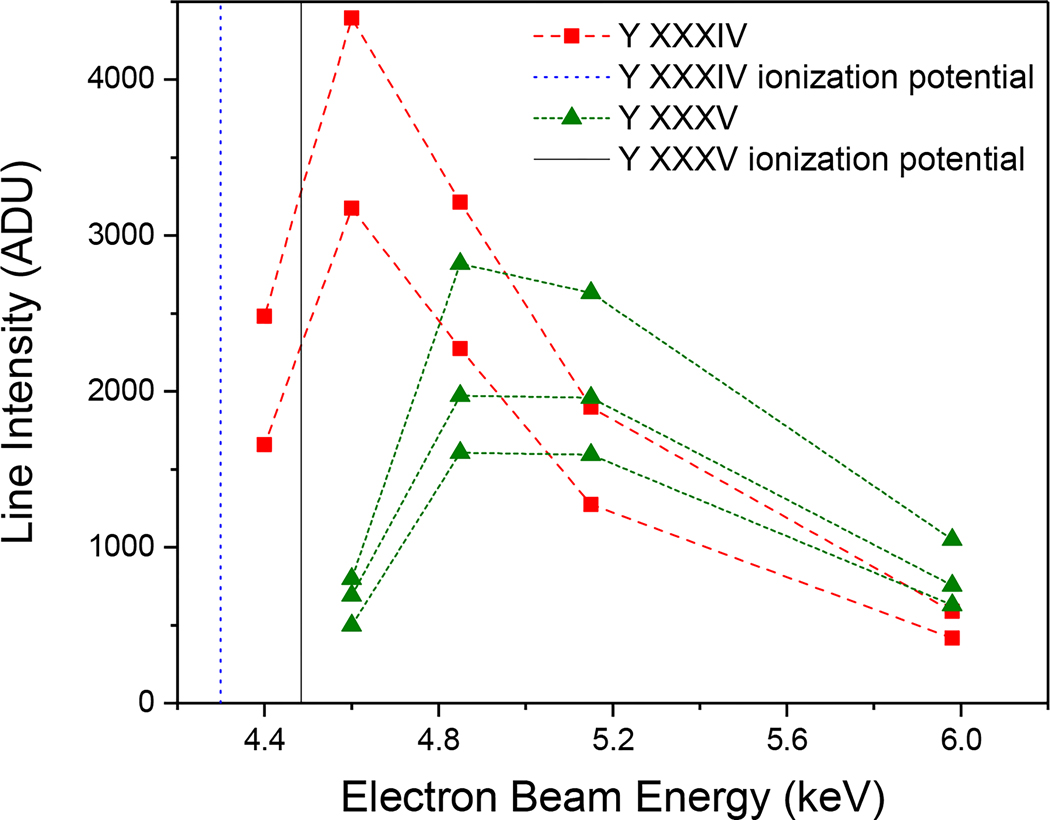
Line intensity plotted as a function of electron beam energy for three Y XXXV lines (green triangles) and two Y XXXIV lines (red squares). The solid (black) and dotted (blue) vertical lines depict the ionization potential of Y XXXV and Y XXXIV, respectively.

In order to help with the identification of lines that were close in wavelength, we considered the evolution of charge states with electron beam energy by plotting the line intensities as a function of the beam energy. The ionization energies of the different charge states determine the minimum beam energy required for the emergence of a particular charge state. For instance, the ionization energy of Y XXXIV is 4.299 keV [64]; hence, a beam energy of 4.299 keV or higher is required to observe spectral lines from Be-like Y XXXV. This gives an idea of the range of charge states one is supposed to observe at a particular beam energy. In addition, lines emitted from the same ionization state usually depend in a similar way on the beam energy. These qualitative dependencies aided the line identification as illustrated in Figure 3. In order to verify these qualitative assumptions, we have used our detailed CR model calculations to make a final assignment based on the line intensity dependences.

## 6. Results and Discussions

**Figure 4.**
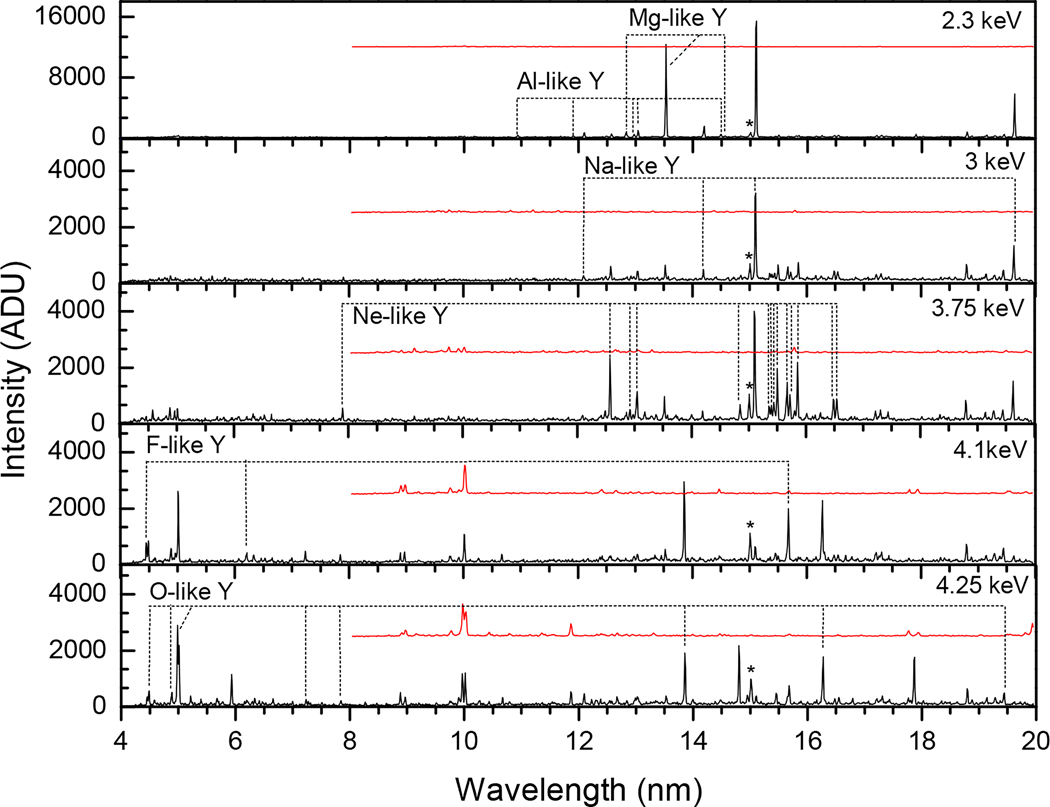
Yttrium spectra at beam energies from 2.30 keV–4.25 keV. The black and red (shifted) spectra correspond to the first and second order Y spectra, respectively. The ∗ marks the impurity coming from oxygen at 15.0099(5) nm.

**Figure 5.**
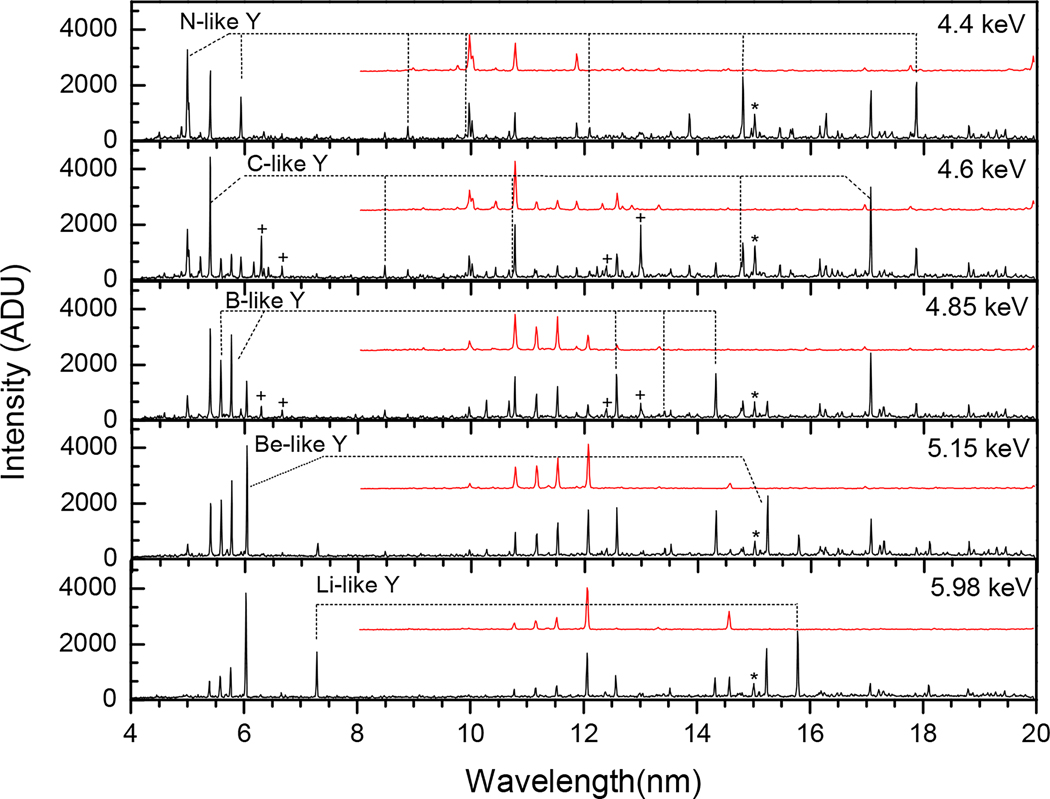
Yttrium spectra at beam energies from 4.40 keV–5.98 keV. The black and red (shifted) spectra correspond to the first and second order Y spectra, respectively. The + marks the Na-like and Mg-like xenon impurities at beam energies of 4.6 keV and 4.85 keV and the ∗ marks the impurity coming from oxygen at 15.0099(5) nm.

Table 1 presents the yttrium lines identified in our experiment for the charge state range between Y XXVII and Y XXXVII. We focused on lines that originate from Δn=0 transitions within the n=2 and n=3 principal quantum number states. Most of the lines are electric dipole (E1) transitions, while a few lines are magnetic dipole (M1) transitions. All M1 lines correspond to transitions within the $2s^{2}2p^{m}$ ground state configurations of different charge states (m=1,2,3,4,5), with the exception of the line at 16.4817(7) nm, which originates from within the excited configuration $2p^{5}3s$ of Ne-like yttrium ion. The energy levels within the ground configuration are close and result in longer wavelength forbidden lines, while the energy levels contributing to the allowed (E1) transitions are further separated and give rise to shorter wavelength lines. Level notations are taken from FAC and are given in jj-coupling. The plus sign stands for the j value of l+1/2, and the minus sign represents the *j* value of l−1/2, where *l* is the orbital angular momentum. As an example, the line at 5.9329(4) nm connects the $2s^{2}2p^{3}$ upper level with a total angular momentum of J=5/2 to the $2s2p^{4}$ of J=3/2. It should be noted that in FAC, the subshells that couple to zero angular momentum are omitted in the notation. As an illustration of this, the $2p^{5}$ configuration is noted as $2p_{+}^{3}$ when both electrons on the 2p_−_ subshell are present and couple to zero joint angular momentum. Yttrium spectra recorded at different energies are shown in Figures 4 and 5, with the identified lines labeled by their isoelectronic sequence.

A total of 59 spectral lines were identified in this work from the Li-like to the Al-like isoelectronic sequences. Of these lines, 38 are new and 21 correspond to previously measured transitions in O-like, F-like, Na-like, Mg-like and Al-like charge states [15,16,21,24–26,29,30,37,43–45,77]. The previously measured transitions are also listed in Table 1 together with their currently measured wavelengths. We observed an O VI line at 15.0099(5) nm in all of the spectra due to impurities in the trap. At 4.60 keV and 4.85 keV drift tube voltages, we also observed impurity lines due to xenon, including a Xe XLII line at 15.0116(7) nm blended with the above-mentioned O VI line. Mg-like Xe XLIII lines were observed at 6.2903(6) nm and 12.9969(9) nm wavelengths, and two Na-like Xe XLIV lines were found at 6.6628(7) nm and 12.3939(7) nm. These lines are listed in the NIST Atomic Spectra Database [7,64] at 6.288(3) nm, 12.993(3) nm, 6.6628(5) nm and 12.394(1) nm, respectively.

**Figure 6.**
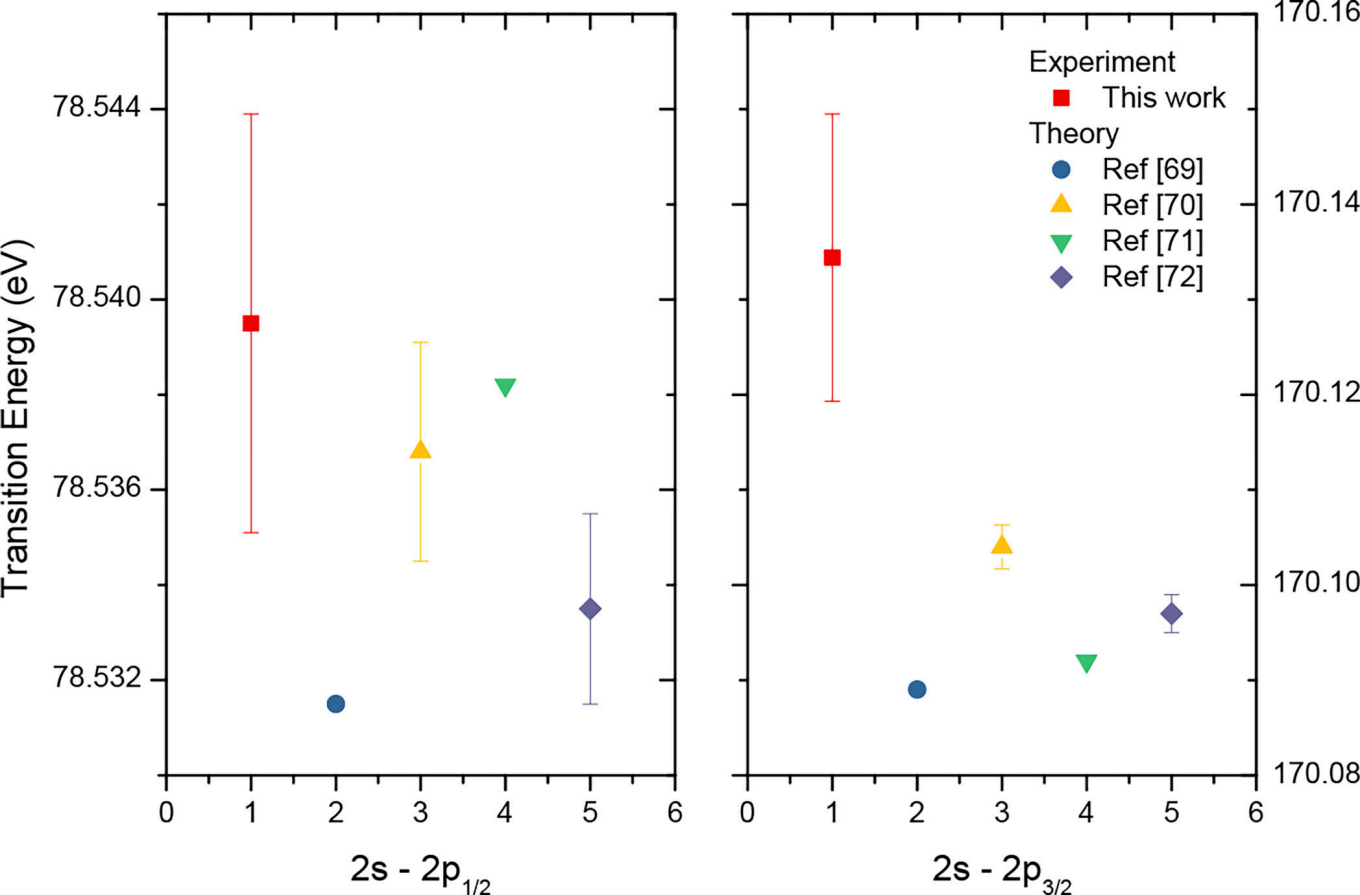
Comparison of measured and calculated Li-like yttrium lines.

Two Li-like yttrium lines were identified at 7.2874(6) nm/170.134(15) eV and 15.7862(9) nm/ 78.5395(44) eV, corresponding to the $\left(2s_{+}\right)-\left(2p_{+}\right)$ and $\left(2p_{+}\right)-\left(2p_{+}\right)$ electric dipole transitions, respectively. The Li-like isoelectronic sequence has been extensively studied both theoretically and experimentally due to its simple electronic structure. Highly accurate ab initio calculations agree with precise experimental results at the high-Z end of the isoelectronic sequence [1,89]. Although recent results are sensitive to higher order QED terms, further developments are expected, especially in the moderately high-Z region where experiments can provide accurate data due to the wavelength range available to grazing incidence EUV spectrometers. Our current relative uncertainty of 57 × 10^−6^ for the wavelength of the $\left(2s_{+}\right)-\left(2p_{-}\right)$ transition shows good agreement with previous high precision calculations [70–72]; however, the $\left(2s_{+}\right)-\left(2p_{+}\right)$ theoretical results [70,72] are slightly outside our relative uncertainty of 82 × 10^−6^ as shown in Figure 6. Upon close examination, a small feature of unidentified origin was found in the low wavelength wings of both Li-like lines. They were taken into account with the inclusion of a second small Gaussian peak in the fits. The reported results and uncertainties reflect the inclusion of these features, and we therefore believe that they are not responsible for the slight disagreement between the theoretical values and our results for the $\left(2s_{+}\right)-\left(2p_{+}\right)$ line.

The two electric dipole Be-like yttrium lines in the measured spectra at 6.0322(5) nm and 15.2336(7) nm correspond to the transitions of ${\left(2s_{+}^{2}\right)}_{0}-{\left(2s_{+},2p_{+}\right)}_{1}$ and ${\left(2s_{+}^{2}\right)}_{0}-{\left(2s_{+},2p_{-}\right)}_{1}$ respectively. Be-like ions are quasi two-electron systems. Therefore, calculations at the level of the precision of the measurement are difficult. Denne et al. [24] predicted these wavelengths in yttrium by fitting the difference between the theoretical and experimental wave numbers and then extrapolating to attain the fine-structure separation. Their predictions of 6.0337(20) nm and 15.2345(20) nm had uncertainties much larger than our measurements. Thus, we provide a considerable increase in the accuracy of the wavelengths.

A similar approach was used by Mrynas et al. [25] for the predictions of the wavelengths of B-like yttrium $\left(2p_{-}\right)-{\left({\left(2s_{+},2p_{-}\right)}_{1},2p_{+}\right)}_{1/2},\left(2p_{-}\right)-{\left({\left(2s_{+},2p_{-}\right)}_{1},2p_{+}\right)}_{3/2}$ and $\left(2p_{-}\right)-\left(2p_{+}\right)$ lines. The obtained respective values were 5.5771 nm, 5.7629 nm and 14.322 nm, but no uncertainties were provided for the fitted wavelength predictions. These results are in a generally good agreement with our observed values of 5.5768(6) nm, 5.7623(6) nm and 14.3234(5) nm. Beyond these three transitions, two additional lines have been identified for B-like yttrium, as shown in Table 1. Out of the five reported transitions, four are E1, and one is an M1 transition.

With an increasing number of electrons, the electronic structure of open shell ions becomes more difficult for theory. However, the experimental wavelength determinations are as accurate as for their simpler structure counterparts. Accurate wavelength results in these ions can provide guidance for further theoretical work for the better understanding of the electron-electron interactions in these systems. Here, we report E1 and M1 transitions for both C-like and N-like yttrium ions.

Behring et al. [77] observed O-like yttrium transitions by irradiating a solid yttrium target with 24 frequency-tripled laser beams. We identified six E1 transitions and two M1 transitions in the same system and provide wavelength values for these in Table 1. The observations of Behring et al. are consistent with our measurements with the exception of the M1 transition ${\left(2p_{+}^{2}\right)}_{2}-{\left(2p_{-},{\left(2p_{+}^{3}\right)}_{3/2}\right)}_{2}$. Our measurement of 13.8581(6) nm for this M1 line is at a shorter wavelength than their predicted wavelength of 13.89(2) nm. The M1 transition at 16.2725(9) nm agrees with their predicted wavelength of 16.28(2) nm [77].

Wavelengths of F-like yttrium lines were measured by Reader et al. [37] using laser produced plasmas. The ${\left(2p_{+}^{3}\right)}_{3/2}-\left(2s_{+}\right)$ and $\left(2p_{-}\right)-\left(2s_{+}\right)$ transitions in these ions were measured to be 4.4496(15) nm and 6.2107(15) nm, respectively. Our results for the same transitions indicated 4.4500(7) nm and 6.2115(14) nm wavelengths and are in good agreement with the previously observed values. Calculations by Feldman et al. [26] reported values of 4.4083 nm and 6.1299 nm that are further away from these measurements than our FAC calculated wavelengths values of 4.417 nm and 6.1454 nm.

The M1 transition ${\left(2p_{+}^{3}\right)}_{3/2}-\left(2p_{-}\right)$ in F-like Y is interesting due to its potential for plasma diagnostics [37,38]. Reader et al. [37] predicted the wavelength by comparing the observed fine-structure intervals with Dirac–Fock calculations and obtained 15.681(12) nm. This is in agreement with our measured value of 15.6801(11) nm.

The ground state of Ne-like ions is a closed shell. However, the low lying excited states have interesting features that have been exploited in many experiments and observations [90]. The level structure has been investigated for use in the diagnosis of astrophysical and laboratory plasmas [91,92] and has been used in soft X-ray laser schemes [93]. We report fourteen E1 and one M1 transitions in Ne-like Y. The Ne-like yttrium lines were identified using the theoretical values from highly accurate RMBPT calculations [68].

In our spectra, we were able to identify four E1 transitions of Na-like yttrium ions. The two most prominent ones are the well-known Na-like ${\text{D}}_{1}\left(3s_{+}\right)-\left(3p_{+}\right)$ and ${\text{D}}_{2}\left(3s_{+}\right)-\left(3p_{-}\right)$ lines. Our measured wavelength values of 15.1037(5) nm and 19.6212(7) nm, respectively, lie within the uncertainty of the 15.1035(10) nm and 19.6215(10) nm measurements by Reader et al. in tokamak plasmas and laser-produced plasmas [16]. Seely et al. [29] reported calculated values of 15.0961 nm and 15.0310 nm for the D_1_ line and 19.6047 nm and 19.4851 nm for the D_2_ line with and without QED corrections, respectively. This illustrates the importance of QED corrections at this level of experimental accuracy. Their fitted values of 15.1033 nm and 19.6219 nm for these transitions are within our experimental uncertainty. Our measured wavelengths also agree well with Blundell’s calculated values of 15.10402(40) nm and 19.6209(7) nm for the D_1_ and D_2_ lines [7]. Gillaspy et al. [7] have pointed out that the accuracy of the measurements in medium-Z to high-Z systems is sensitive to the finite nuclear size correction in the otherwise calculable QED terms. This illustrates the importance of these transitions in studies at the interface of atomic and nuclear physics.

Our goal in these studies was the identification of lines in the L-shell ion states of yttrium in the EUV region. The few M-shell charge states we report here appeared at the low energy end of our systematic scans. The highest isoelectronic sequences investigated here were those for Mg and Al. In Mg-like yttrium, we report the observation of three E1 lines that have been previously measured by Ekberg et al. [21], Sugar et al. [15] and Reader et al. [44]. Similarly, the five Al-like yttrium E1 lines that we identified were previously measured by Ekberg et al. [43] and Sugar et al. [30]. The slight disagreement with some of the previous measurements might be due to weak lines and blends with other line features.

## 7. Diagnostically Important M1 Transitions

Among the 59 identified yttrium lines listed in Table 1, 10 lines are due to forbidden M1 transitions. States that decay via M1 transitions have a different dependence on collisional depopulation from states with E1 transitions. Thus, the corresponding intensity ratios of M1 to E1 lines are sensitive to the electron densities and temperatures, thereby making them potential candidates for plasma diagnostics. To analyze the feasibility of this, calculations with the CR modeling code NOMAD were performed for Maxwellian electron energy distribution plasmas with electron densities ranging from 10^12^ cm^−3^–10^20^ cm^−3^ and temperatures from 1500 eV–6000 eV, which provide the largest abundance of the ions.

**Figure 7.**
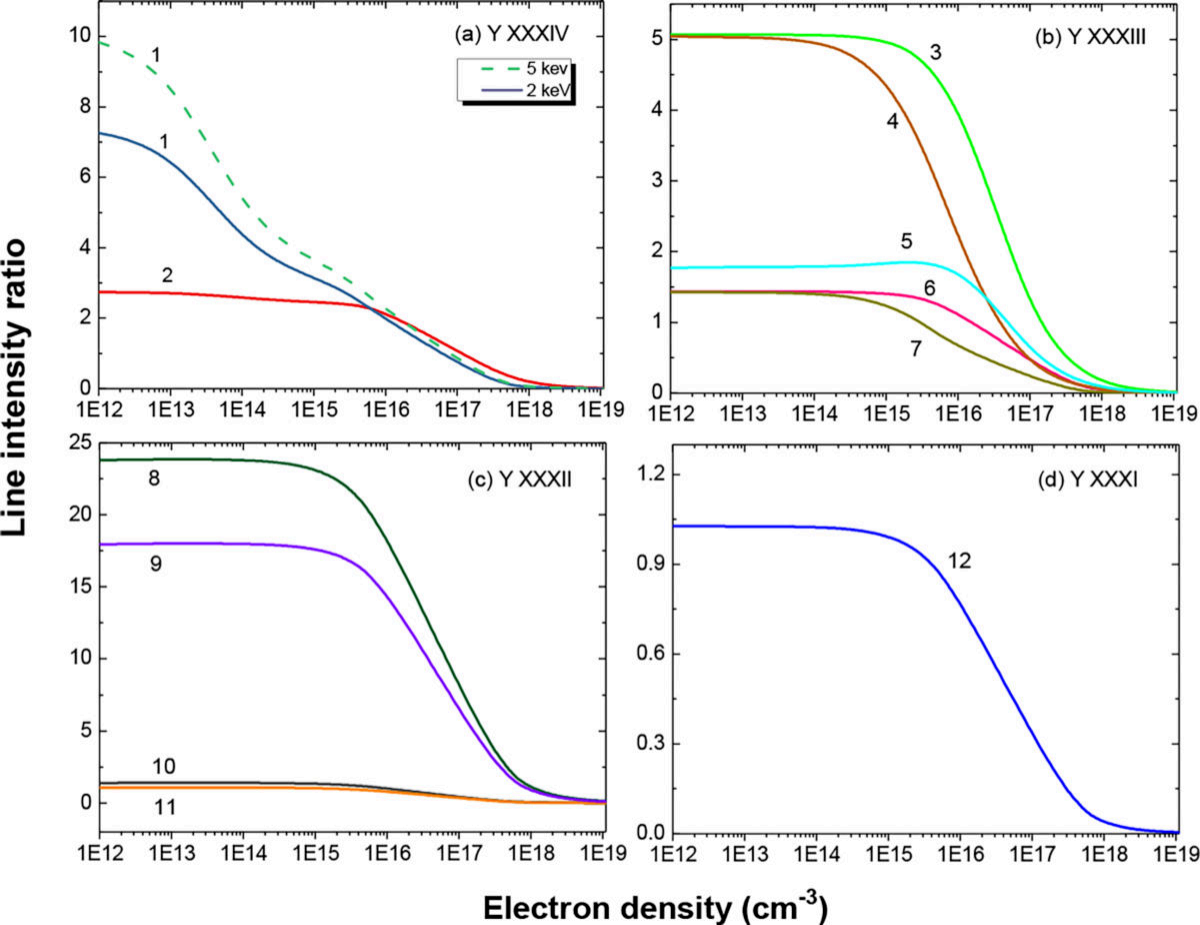
Density-sensitive line ratios for (**a**) C-like Y XXXIV, (**b**) N-like Y XXXIII, (**c**) O-like Y XXXII and (**d**) F-like Y XXXI. The number labels correspond to the line ratios: (1) 17.0632(7) nm/11.1236(9) nm at 5-keV electron energy (dash) and 2-keV electron energy (solid), (2) 17.0632(7) nm/8.4792(19) nm, (3) 17.8665(6) nm/9.9054(10) nm, (4) 14.8036(5) nm/9.9054(10) nm, (5) 12.0926(6) nm/9.9054(10) nm, (6) 17.8665(6) nm/8.8822(7) nm, (7) 14.8036(5) nm/8.8822(7) nm, (8) 13.8581(6) nm/19.4383(8) nm, (9) 16.2725(9) nm/19.4383(8) nm, (10) 16.2725(9) nm/7.2352(8) nm, (11) 13.8581(6) nm/7.2352(8) nm and (12) 15.6801(11) nm/6.21115(14) nm.

The most sensitive intensity ratios for the spectral lines of ${\text{Y}}^{30+}-{\text{Y}}^{33+}$ ions are presented in Figure 7. The figure provides examples for sensitivity to the electron density $\left(n_{e}\right)$ in the range of 10^12^ cm^−3^–10^19^ cm^−3^. At low densities, radiative rates for both forbidden and allowed transitions are much stronger than the collisional rates, and therefore, no dependence on electron density arises. However, at higher densities, collisional quenching dominates radiative decay for forbidden lines, and the line intensity ratios become sensitive to Figure 7. The figure provides examples for sensitivity to the electron density $n_{e}$.

The five C-like yttrium lines listed in Table 1 include an M1 transition at a 17.0632(7) nm wavelength. The ratio of this M1 line to the E1 line at 8.4792(19) nm varies by a factor of 45 or less in the electron density range of 10^15^ cm^−3^–10^18^ cm^−3^. The line intensity ratio I(17.0632(7))/I(11.1236(9)) varies by more than two orders of magnitude for the same range of electron density. This line ratio also shows dependence on the electron temperature at lower densities as illustrated in Figure 7a. The line ratio of the M1 line at 17.0632(7) nm to another line at the FAC wavelength of 12.3869 nm shows a similar dependence on electron density and temperature because both of the E1 transitions at 11.1236(9) nm and 12.3869 nm arise from the same upper level decaying to the second and third energy levels, respectively. The line at 12.3869 nm, which is about half of the intensity of line at 11.1236(9) nm, could not be resolved due to strong blend with a Na-like Xe line at 12.3939(7) nm. However, at other plasma conditions with no Xe, this line should be easily resolved.

**Figure 8.**
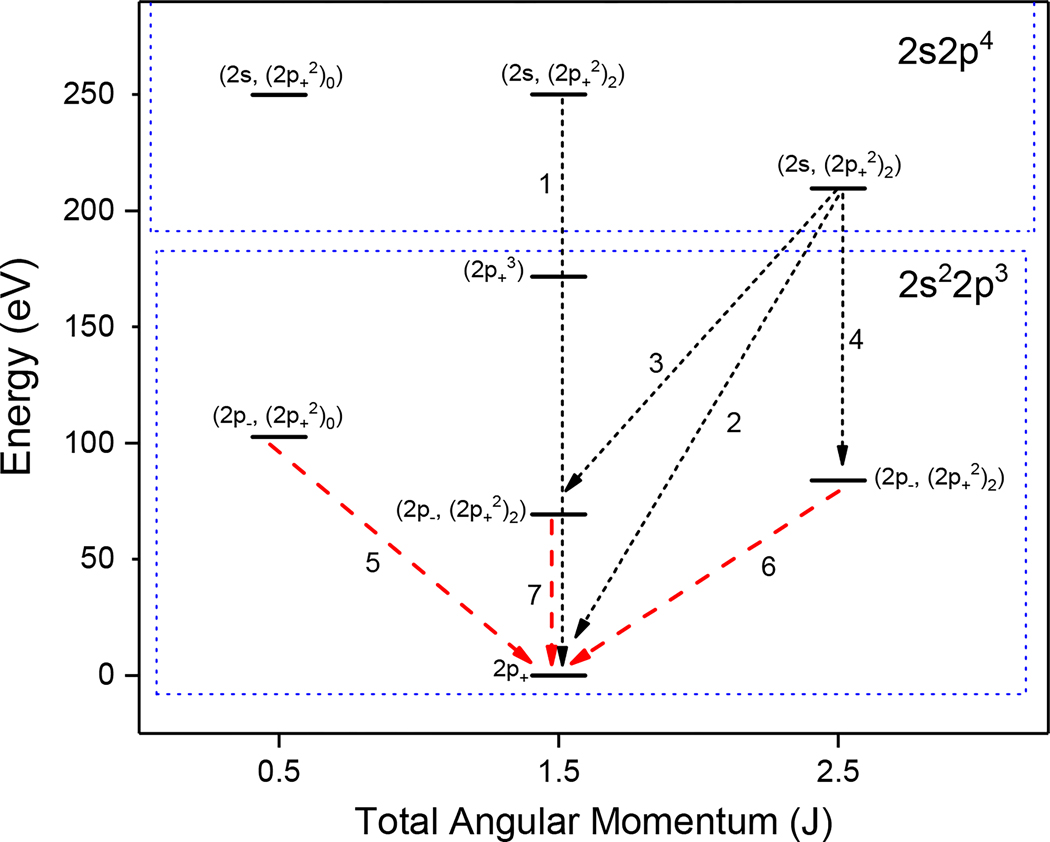
Partial Grotrian diagram of the ground state and first lowest excited configurations of N-like yttrium. The number labels in increasing order from 1–7 correspond to the lines at 4.9858(6) nm, 5.9329(4) nm, 8.8822(7) nm, 9.9054(10) nm, 12.0926(6) nm, 14.8036(5) nm and 17.8665(6) nm, respectively. Only three of the energy levels for the $2s2p^{4}$ configuration are shown. The dashed red lines correspond to magnetic dipole (M1) transitions, and the dotted black lines correspond to electric dipole (E1) transitions.

Among the seven identified N-like yttrium lines, four of the lines arise from E1 transitions, and three arise from M1 transitions. The ratios of the M1/E1 intensity show a dependence on $n_{e}$ between 10^15^ cm^−3^ and 10^18^ cm^−3^ of a maximum of two orders of magnitude. According to our FAC calculations, the transition probability of the M1 line at 14.8036(5) nm is 6.75 × 10^5^ s^−1^ compared to the transition probability of 3.9 × 10^6^ s^−1^ for the M1 line at 12.0926(6) nm and 3.7 × 10^6^ s^−1^ for the M1 line at 17.8665(6) nm. This explains why the ratios I(14.8036(5))/I(9.9054(10)) and I(14.8036(5))/I(8.8822(7)) start decreasing at densities of 10^15^ cm^−3^, whereas the intensity ratios of the M1 line at 12.0926(6) nm to the E1 lines and the M1 line at 17.8665(6) nm to the E1 lines only start to fall off at densities of 10^16^ cm^−3^ and higher. These transitions are illustrated in the Grotrian diagram shown in Figure 8.

A closer look at the the density dependence of M1/E1 ratios in N-like ions gives an insight into the population scheme of allowed and metastable upper levels. For instance, let us take the population and depopulation channels of the metastable level in the ground state configuration $2p^{3}$ that is the upper level of the 17.8665(6) nm M1 transition and a $2s2p^{4}$ excited state level that is the origin of three E1 transitions (5.9329(4) nm, 8.8822(7) nm and 9.9054(10) nm wavelengths). At a temperature of 1500 eV, the metastable level 2*p*^3^ at a lower density of 10^12^ cm^−3^ is depopulated by radiative decay with 99.96% probability. At a higher density of 10^16^ cm^−3^, the depopulation is 73% by collisional excitation to higher levels, 3% by collisional deexcitation to a lower ground state level, and only a 21% probability remains for radiative decay. At an even more elevated electron density of 10^18^ cm^−3^, the level is depopulated mostly by collisional excitation with nearly 94% probability, leaving 4% to collisional deexcitation, 1% to radiative recombination and a negligible probability (0.27%) to radiation. The $2s2p^{4}$ excited level is depopulated 100% by radiative decay at 10^12^ cm^−3^ and 10^16^ cm^−3^, and the probability only slightly lowers to 99.6% at 10^18^ cm^−3^. This means that the ratio of the intensity of the $2s2p^{4}$ E1 transitions to that of the $2p^{3}$ M1 transition shows a strong variation with the electron density.

Out of the eight O-like yttrium lines, we observed two that originate from M1 transitions at 13.8581(6) nm and 16.2725(9) nm. The intensity ratios between the M1 and E1 transitions vary by more than an order of magnitude in the density ranges we investigated. For instance, the intensity ratio I(13.8581(6))/I(19.4383(8)) changes by a factor of 67 for densities ranging from 10^15^ cm^−3^–10^18^ cm^−3^.

For the one M1 and two E1 lines in F-like Y, an order of magnitude variation is seen for the intensity ratio of M1 line at 15.6801(11) nm to the E1 line at 6.2115(14) nm.

## 8. Conclusions

New and previously-measured EUV lines in L-shell ions along with transitions in a few M-shell ions of highly charged yttrium were observed. The measurements were performed with an electron beam ion trap, and spectral lines were recorded in the wavelength region of 4 nm–20 nm. The experimental uncertainties were combinations of statistical and systematic uncertainties that included sources with calibration origins and uncertainties from unresolved blends. The total uncertainties ranged between 0.0004 nm and 0.0020 nm. Line identifications were inferred from comparisons with spectra simulated from the collisional-radiative model NOMAD based on a non-Maxwellian distribution designed for EBIT-like environments. For Ne-like Y ions, a better agreement between theory and experiment was found using relativistic many-body perturbation theory (RMBPT) [68]. Several of the identified forbidden M1 transitions were found to be potentially useful for density diagnostics of laboratory, fusion and astrophysical plasmas.

## Figures and Tables

**Table 1.** The table below presents the list of the yttrium lines identified ${\text{Y}}^{26+}-{\text{Y}}^{36+}$. Previous measurements and calculations are reported as well. Isoelectronic sequence is abbreviated as Seq., configuration is abbreviated as Config., and the level number is abbreviated as No.

Ion Charge	Seq.	Type		Lower Level			Upper Level		Experimental Wavelength (nm)		Theoretical Wavelength (nm)	
			No.	Config.	${\text{Term}}_{J}$	No.	Config.	${\text{Term}}_{J}$	This Work	Previous Work	This Work	Previous Work
36	Li	E1	1	2s	$2s_{+}$	2	2p	$2p_{+}$	7.2874(6)		7.2771	7.2893 [69] 7.2887(1) [70] 7.2892 [71] 7.2890(1) [72] 7.2888 [73]
36	Li	E1	1	2s	$2s_{+}$	3	2p	$2p_{-}$	15.7862(9)		15.7139	15.7878 [69] 15.7868(5) [70] 15.7865 [71] 15.7874(4) [72] 15.7867 [73]
35	Be	E1	1	$2s^{2}$	$({2s}_{+}^{2}{)}_{0}$	6	2s2p	$(2s_{+},\;2p_{+}{)}_{1}$	6.0322(5)		6.0098	6.0337(20) ^f^ [24] 6.0283 [74]
35	Be	E1	1	$2s^{2}$	$({2s}_{+}^{2}{)}_{0}$	3	2s2p	$(2s_{+},\;2p_{-}{)}_{1}$	15.2336(7)		15.1907	15.2345(20) ^f^ [24] 15.2302 [74]
34	B	E1	1	2p	$2p_{-}$	7	$2s2p^{2}$	${\left({\left(2s_{+},\;2p_{-}\right)}_{1},\;2p_{+}\right)}_{1/2}$	5.5768(6)		5.5310	5.5771 ^f^ [25]
34	B	E1	1	2p	$2p_{-}$	6	$2s2p^{2}$	${\left({\left(2s_{+},\;2p_{-}\right)}_{1},\;2p_{+}\right)}_{3/2}$	5.7623(6)		5.7254	5.7629 ^f^ [25]
34	B	E1	1	2p	$2P_{-}$	3	$2s2p^{2}$	$2s_{+}$	12.5693(6)		12.5372	
34	B	E1	2	2p	$2p_{+}$	5	$2s2p^{2}$	${\left({\left(2s_{+},\;2p_{-}\right)}_{1},\;2p_{+}\right)}_{5/2}$	13.4185(8)		13.3700	
34	B	M1	1	2p	$2p_{-}$	2	2p	$2p_{+}$	14.3234(5)		14.3363	14.321 [75] 14.322 ^f^ [25]
33	C	E1	1	$2p^{2}$	$({2p}_{-}^{2}{)}_{0}$	7	$2s2p^{3}$	$(2s_{+},\;2p_{+}{)}_{1}$	5.3878(5)		5.3571	
33	C	E1	3	$2p^{2}$	$(2p_{-},\;2p_{+}{)}_{2}$	7	$2s2p^{3}$	$(2s_{+},\;2p_{+}{)}_{1}$	8.4792(19) ^b^		8.4071	
33	C	E1	2	$2p^{2}$	$(2p_{-},\;2p_{+}{)}_{1}$	5	$2s2p^{3}$	$(2p_{+},\;2p_{+}{)}_{2}$	11.1236(9) ^b^		11.1132	
33	C	M1	1	$2p^{2}$	$({2p}_{-}^{2}{)}_{0}$	3	$2p^{2}$	$(2p_{-},\;2p_{+}{)}_{2}$	14.7700(10)		14.7668	
33	C	M1	1	$2p^{2}$	$({2p}_{-}^{2}{)}_{0}$	2	$2p^{2}$	$(2p_{-},\;2p_{+}{)}_{1}$	17.0632(7)		17.1036	17.0625 [76] 17.0558 [76]
32	N	E1	1	$2p^{3}$	$2p_{+}$	8	$2s2p^{4}$	${\left(2s_{+},{\left(2p_{+}^{2}\right)}_{2}\right)}_{3/2}$	4.9858(6)		4.9593	
32	N	E1	1	$2p^{3}$	$2p_{+}$	6	$2s2p^{4}$	${\left(2s_{+},{\left(2p_{+}^{2}\right)}_{2}\right)}_{5/2}$	5.9329(4)		5.9151	
32	N	E1	2	$2p^{3}$	${\left(2p_{-},{\left(2p_{+}^{2}\right)}_{2}\right)}_{3/2}$	6	$2s2p^{4}$	${\left(2s_{+},{\left(2p_{+}^{2}\right)}_{2}\right)}_{5/2}$	8.8822(7)		8.8358	
32	N	E1	3	$2p^{3}$	${\left(2p_{-},{\left(2p_{+}^{2}\right)}_{2}\right)}_{5/2}$	6	$2s2p^{4}$	${\left(2s_{+},{\left(2p_{+}^{2}\right)}_{2}\right)}_{5/2}$	9.9054(10)		9.8612	
32	N	M1	1	$2p^{3}$	$2p_{+}$	4	$2p^{3}$	${\left(2p_{-,}{\left(2p_{+}^{2}\right)}_{0}\right)}_{1/2}$	12.0926(6)		12.0717	
32	N	M1	1	$2p^{3}$	$2p_{+}$	3	$2p^{3}$	${\left(2p_{-},{\left(2p_{+}^{2}\right)}_{2}\right)}_{5/2}$	14.8036(5)		14.7819	
32	N	M1	1	$2p^{3}$	$2p_{+}$	2	$2p^{3}$	${\left(2p_{-,}{\left(2p_{+}^{2}\right)}_{2}\right)}_{3/2}$	17.8665(6)		17.8947	
31	O	E1	1	$2p^{4}$	${\left(2p_{+}^{2}\right)}_{2}$	7	$2s2p^{5}$	${\left(2s_{+},{\left(2p_{+}^{3}\right)}_{3/2}\right)}_{1}$	4.4854(8)	4.4857(15) [77]	4.4567	
31	O	E1	2	$2p^{4}$	${\left(2p_{+}^{2}\right)}_{0}$	7	$2s2p^{5}$	${\left(2s_{+},{\left(2p_{+}^{3}\right)}_{3/2}\right)}_{1}$	4.8871(12)	4.8882(15) [77]	4.8569	
31	O	E1	1	$2p^{4}$	${\left(2p_{+}^{2}\right)}_{2}$	6	$2s2p^{5}$	${\left(2s_{+},{\left(2p_{+}^{3}\right)}_{3/2}\right)}_{2}$	5.0103(5)	5.0085(15) [77]	4.9828	
31	O	E1	3	$2p^{4}$	${\left(2p_{-},{\left(2p_{+}^{3}\right)}_{3/2}\right)}_{1}$	6	$2s2p^{5}$	${\left(2s_{+},{\left(2p_{+}^{3}\right)}_{3/2}\right)}_{2}$	7.2352(8)	7.2356(15) [77]	7.1754	
31	O	E1	4	$2p^{4}$	${\left(2p_{-,},{\left(2p_{+}^{3}\right)}_{3/2}\right)}_{2}$	6	$2s2p^{5}$	${\left(2s_{+},{\left(2p_{+}^{3}\right)}_{3/2}\right)}_{2}$	7.8430(8)		7.7848	
31	O	M1	1	$2p^{4}$	${\left(2p_{+}^{2}\right)}_{2}$	4	$2p^{4}$	${\left(2p_{-,}{\left(2p_{+}^{3}\right)}_{3/2}\right)}_{2}$	13.8581(6)		13.8442	13.89(2) [77]
31	O	M1	1	$2p^{4}$	${\left(2p_{+}^{2}\right)}_{2}$	3	$2p^{4}$	${\left(2p_{-},{\left(2p_{+}^{3}\right)}_{3/2}\right)}_{1}$	16.2725(9)		16.307	16.28(2) [77]
31	O	E1	16	$2p^{3}3p$	${\left(2p_{+},\;3p_{+}\right)}_{3}$	32	$2p^{3}3d$	${\left(2p_{+},\;3d_{+}\right)}_{4}$	19.4383(8)		19.4639	
30	F	E1	1	$2p^{5}$	${\left(2p_{+}^{3}\right)}_{3/2}$	3	2s	$2s_{+}$	4.4500(7)	4.4496(15) [37]	4.417	4.4083 [26] 4.4486 ^f^ [26] 4.4492 [39]
30	F	E1	2	$2p^{5}$	$2p_{-}$	3	2s	$2s_{+}$	6.2115(14) ^b^	6.2107(15) [37]	6.1454	6.1299 [26] 6.2109 ^f^ [26]
30	F	M1	1	$2p^{5}$	${\left(2p_{+}^{3}\right)}_{3/2}$	2	$2p^{5}$	$2p_{-}$	15.6801(11)		15.7043	15.681(12) ^f^ [37] 15.654(5)^f^ [78] 15.678 ^f^ [26] 15.678(12) [79] 15.71 [38] 15.6826 [39] 15.685 ^f^ [80]
29	Ne	El	3	$2p^{5}3s$	$\left({\left(2p_{+}^{3}\right)}_{3/2}\right),{3s_{+})}_{1}$	20	$2p^{5}3p$	${\left(2p_{-},\;3p_{-}\right)}_{0}$	7.8983(8)		7.9003 ^a^	7.914 ^f^ [81]
29	Ne	El	3	$2p^{5}3s$	$\left({\left(2p_{+}^{3}\right)}_{3/2}\right),{3s_{+})}_{1}$	11	$2p^{5}3p$	${\left({\left(2p_{+}^{3}\right)}_{3/2},\;3p_{+}\right)}_{0}$	12.5743(7)		12.5696 ^a^	12.576 ^f^ [81]
29	Ne	El	12	$2p^{5}3p$	${\left(2p_{-},\;3p_{-}\right)}_{1}$	24	$2p^{5}3d$	${\left(2p_{-},\;3d_{-}\right)}_{2}$	12.9238(8)		12.9267 ^a^	
29	Ne	El	5	$2p^{5}3p$	${\left({\left(2p_{+}^{3}\right)}_{3/2},\;3p_{-}\right)}_{2}$	15	$2p^{5}3d$	${\left({\left(2p_{+}^{3}\right)}_{3/2},\;3d_{-}\right)}_{3}$	13.047l(8)		13.0550 ^a^	
29	Ne	El	2	$2p^{5}3s$	${\left({\left(2p_{+}^{3}\right)}_{3/2},\;3s_{+}\right)}_{2}$	10	$2p^{5}3p$	${\left({\left(2p_{+}^{3}\right)}_{3/2},\;3p_{+}\right)}_{2}$	14.8480(7)		14.8389 ^a^	
29	Ne	E1	10	$2p^{5}3p$	${\left({\left(2p_{+}^{3}\right)}_{3/2},\;3p_{+}\right)}_{2}$	22	$2p^{5}3d$	${\left({\left(2p_{+}^{3}\right)}_{3/2},\;3d_{+}\right)}_{3}$	15.3559(10)		15.3587 ^a^	
29	Ne	El	19	$2p^{5}3p$	${\left(2p_{-},\;3p_{+}\right)}_{2}$	26	$2p^{5}3d$	${\left(2p_{-},\;3d_{+}\right)}_{3}$	15.3945(10)		15.3972 ^a^	
29	Ne	El	l8	$2p^{5}3p$	${\left(2p_{-},\;3p_{+}\right)}_{1}$	25	$2p^{5}3d$	${\left(2p_{-},\;3d_{+}\right)}_{2}$	15.4387(10)		15.4444 ^a^	
29	Ne	El	3	$2p^{5}3s$	${\left({\left(2p_{+}^{3}\right)}_{3/2},\;3s_{+}\right)}_{1}$	10	$2p^{5}3p$	${\left({\left(2p_{+}^{3}\right)}_{3/2},\;3p+\right)}_{2}$	15.4902(18) ^b^	15.497(15) [82] 15.50 [83]	15.4882 ^a^	15.503 ^f^ [81] 15.50 [84]
29	Ne	El	9	$2p^{5}3s$	${\left(2p_{-},\;3s_{+}\right)}_{1}$	20	$2p^{5}3p$	${\left(2p_{-},\;3p_{-}\right)}_{0}$	15.5024(8) ^b^		15.4904 ^a^	15.498^f^[81]
29	Ne	El	6	$2p^{5}3p$	${\left({\left(2p_{+}^{3}\right)}_{3/2},\;3p_{+}\right)}_{3}$	l7	$2p^{5}3d$	${\left({\left(2p_{+}^{3}\right)}_{3/2},\;3d_{+}\right)}_{4}$	15.6711(10)		15.6769 ^a^	
29	Ne	El	9	$2p^{5}3s$	${\left(2p_{-},\;3s_{+}\right)}_{1}$	19	$2p^{5}3p$	$(2p_{-},\;3p_{+}{)}_{2}$	15.7208(7)	15.714(15) [82] 15.7l [83]	15.7085^a^	15.723^f^ [81] 15.7l [84]
29	Ne	El	2	$2p^{5}3s$	${\left({\left(2p_{+}^{3}\right)}_{3/2},\;3s_{+}\right)}_{2}$	6	$2p^{5}3p$	${\left({\left(2p_{+}^{3}\right)}_{3/2},\;3p_{+}\right)}_{3}$	15.8537(7)		15.8455 ^a^	
29	Ne	Ml	3	$2p^{5}3s$	${\left({\left(2p_{+}^{3}\right)}_{3/2},\;3s_{+}\right)}_{1}$	8	$2p^{5}3s$	${\left((2p_{-},\;3p_{+}\right)}_{2}$	16.4817(7)		l6.4843 ^a^	
29	Ne	El	3	$2p^{5}3s$	${\left({\left(2p_{+}^{3}\right)}_{3/2},\;3s_{+}\right)}_{1}$	7	$2p^{5}3p$	${\left({\left(2p_{+}^{3}\right)}_{3/2},\;3p_{+}\right)}_{1}$	l6.5411(8)	l6.537(15) [82]	l6.5488 ^a^	l6.542 ^f^ [81] l6.463 [85] l6.484 [85]
28	Na	El	2	3p	$3p_{-}$	4	3d	$3d_{-}$	12.0979(8)	12.098(20) [16]	12.1353	12.09248 [86] 12.0993(7) ^f^ [45]
28	Na	El	3	3p	$3p_{+}$	5	3d	$3d_{+}$	14.1938(7)	14.1938(6) [16]	14.2458	14.l873 [86] 14.l959(7) ^f^ [45]
28	Na	E1	1	$3s^{2}$	$3s_{+}$	3	3p	$3p_{+}$	15.1037(5)	15.1035(10) [16]	15.0542	15.1038 [55] 15.10402(40) [7] 15.1033 ^f^ [29] 15.1038(7) ^f^ [45] 15.0658 [86]
28	Na	E1	1	$3s^{2}$	$3s_{+}$	2	3p	$3p_{-}$	19.6212(7)	19.6215(10) [16]	19.5175	19.6199 [55] 19.6209(7) [7] 19.6219 ^f^ [29] 19.6213(7) [45]
27	Mg	E1	5	3s3p	${\left(3s_{+},\;3p_{+}\right)}_{1}$	14	3s3d	${\left(3s_{+},\;3d_{+}\right)}_{2}$	12.8333(9)	12.8352(10) [21] 12.8349(5) [15] 12.8301(15) [44]	12.7875	
27	Mg	E1	1	$3s^{2}$	${\left(3s_{+}^{2}\right)}_{0}$	5	3s3p	$(3s_{+}3p_{+}{)}_{1}$	13.5276(5)	13.5279(10) [21] 13.5283(5) [15] 13.5216(15) [44]	13.4437	13.5276 [87] 13.5213 [88]
27	Mg	E1	3	3s3p	${\left(3s_{+},\;3p_{-}\right)}_{1}$	7	$3p^{2}$	${\left(3p_{-},\;3p_{+}\right)}_{2}$	14.5650(20) ^b^	14.5603(10) [21]	14.528	
26	Al	E1	1	3p	$3p_{-}$	10	$3s3p^{2}$	${\left(3s_{+},{\left(3p_{+}^{2}\right)}_{0}\right)}_{3/2}$	10.9388(15) ^w, b^	10.9391(20) [30] 10.9413(10) [43]	10.8578	
26	Al	E1	2	3p	$3p_{+}$	11	3s3d	$3d_{-}$	11.9072(12) ^w, b^	11.9131(20) [30] 11.9110(10) [43]	11.8248	
26	Al	E1	2	3p	$3p_{+}$	10	$3s3p^{2}$	${\left(3s_{+},{\left(3p_{+}^{2}\right)}_{2}\right)}_{3}$	12.9717(11) ^w^	12.9729(20) [30] 12.9745(10) [43]	12.852	
26	Al	E1	1	3p	$3p_{-}$	8	$3s3p^{2}$	$((3s_{+},\;3p_{-}{)}_{1}),\;3p_{+}{)}_{1/2}$	13.0401(8)	13.0416(20) [30] 13.0417(10) [43]	12.9265	
26	Al	E1	1	3p	$3p_{-}$	6	$3s3p^{2}$	$((3s_{+},\;3p_{-}{)}_{1}),\;3p_{+}{)}_{3/2}$	14.4883(8) ^w^	14.4914(20) [30] 14.4910(10) [43]	14.4675	

a Wavelength from Safronova et al.’s calculation [68]

b blended with other line feature

w weak lines; and

f fitted values.
